# Gone for good? An online survey of emigrant health professionals using Facebook as a recruitment tool

**DOI:** 10.1186/s12960-016-0130-y

**Published:** 2016-06-30

**Authors:** Sara McAleese, Barbara Clyne, Anne Matthews, Ruairí Brugha, Niamh Humphries

**Affiliations:** Department of Epidemiology and Public Health Medicine, Royal College of Surgeons, Dublin, Ireland; Department of General Practice, Royal College of Surgeons, Dublin, Ireland; School of Nursing and Human Sciences, Dublin City University, Dublin, Ireland

**Keywords:** Doctors, Emigration, Health workforce planning, Midwives, Nurses, Online survey

## Abstract

**Background:**

Health professionals, particularly doctors, nurses and midwives, are in high demand worldwide. Therefore, it is important to assess the future plans and likelihood of return of emigrating health professionals. Nevertheless, health professionals are, by definition, a difficult population to track/survey. This exploratory study reports on the migration intentions of a sample of doctors, nurses and midwives who had emigrated from Ireland, a high-income country which has experienced particularly high outward and inward migration of health professionals since the year 2000.

**Methods:**

Health professionals who had emigrated from Ireland were identified via snowball sampling through Facebook and invited to complete a short online survey composed of closed and open response questions.

**Results:**

A total of 388 health professionals (307 doctors, 73 nurses and 8 midwives) who had previously worked in Ireland completed the survey. While over half had originally intended to spend less than 5 years in their destination country at the time of emigration, these intentions changed over time, with the desire to remain abroad on a permanent basis increasing from 10 to 34 % of doctor respondents. Only a quarter of doctors and a half of nurses and midwives intended to return to practice in Ireland in the future.

**Conclusions:**

The longer health professionals remain abroad, the less likely they are to return to their home countries. Countries should focus on the implementation of retention strategies if the ‘carousel’ of brain drain is to be interrupted. This would allow source countries to benefit from their investments in training health professionals, rather than relying on international recruitment to meet health system staffing needs. Improved data collection systems are also needed to track the migratory patterns and changing intentions of health professionals. Meanwhile, social networking platforms offer alternative methods of filling this information gap.

## Background

### Health professional migration

Health professional (doctors, nurses and midwives) migration has been steadily increasing over the last decade and continues to rise [1]. Source countries (commonly the country of health professional training) from which health professionals traditionally emigrate are low- and middle-income countries (LMICs), whereas destination countries tend to be high-income countries (HICs) [2]; nevertheless, emigration also occurs both within LMICs and within HICs.

### Challenges in monitoring health professional migration

Health professional migration represents a brain drain of highly skilled and qualified individuals [3]. The financial loss due to the emigration of locally-trained doctors from African source countries alone has been estimated at over $2 billion [4]. Additional costs include the resultant shortages and the burden of inappropriately or less qualified health workers on which source country communities must rely on for healthcare. Consequently, the cornerstone of the WHO’s Global Code of Practice on the International Recruitment of Health Personnel (WHO Global Code) [5] is that countries should achieve health workforce self-sufficiency, which requires not only the generation of sufficient numbers of health professionals, but also implementation of policies and incentives to enhance their retention [6].

Unmonitored, health professional mobility (inward and outward migration) can undermine a country’s health workforce policy objectives [7]. Therefore, the starting point for health workforce planning is good information [8]. However, only 36 countries worldwide keep statistical records of health personnel whose initial qualification was obtained in a foreign country, and only 27 countries undertake research on health personnel migration [9]. Furthermore, data collected on health professionals is generally of poor quality [4]. Therefore, improving data collection on health worker migration has been declared a priority by the Organisation for Economic Co-operation and Development [10]. Ireland is beginning to improve upon and coordinate the data it collects concerning the migration of health professionals through the design of a minimum dataset as part of its commitment under the WHO Global Code [5]. However, the reality continues to be that countries struggle with out-of-date and incomplete datasets on the migration, whereabouts and countries of origin of health professionals [7].

While some countries, such as Ireland, are beginning to achieve better surveillance of the inward migration of health professionals through research [11–15] and annual monitoring by professional councils [16, 17], the outward migration of health professionals is more difficult to monitor and measure. Ireland, like most source countries, is not aware of the number of doctors emigrating from its workforce annually or where they emigrate to. In the case of nurses/midwives, the intended destination country is recorded through verification requests; nevertheless, uncertainties persist regarding the extent to which intentions translate to actual emigration. Measuring migration intentions has been found to be a predictor of subsequent migration [18, 19], and is a good proxy considering the limitations of generating longitudinal data to empirically test the link between intentions and actual behaviour. While the need for data on the numbers and destinations of health professionals emigrating is greater in the source country – it can only be easily collected in the destination countries where there is less need and incentive to do so.

Clearly, effective retention mechanisms are as important in HICs as they are in LMICs because of the ripple effect of international recruitment (usually from LMICs), whereby HICs recruit from LMICs to fill resultant health workforce gaps. This dimension of Ireland’s outlying position in global health workforce migration – the large-scale emigration of domestically-trained doctors, nurses and midwives – has hitherto been under-researched (like elsewhere) compared to the published research on the resultant inward migration of foreign-trained doctors and nurses to Ireland [11–15]. Such research is of much more than academic importance for countries losing their health professionals in large numbers considering the need to monitor the scale and types of professionals leaving, to be able to learn lessons from the health systems and health workforce practices of the destination countries and, above all, to be able to locate and learn from these emigrant doctors and nurses. Of particular interest are the future intentions of migrant health professionals and their interest in returning to the country in which they trained [10].

### Health professional emigration from Ireland

By 2013, Ireland had largely achieved the policy goal of graduating sufficient doctors to meet its domestic needs through a doubling of the medical school intake of Irish and European Union students from 305 to 730 annually [20]. However, vacancies at all levels, from junior hospital training grades to hospital consultants, continue to increase [21, 22]. As a result, there has been no reduction in Ireland’s reliance on the recruitment of international medical graduates, who have accounted for 30–35 % of all doctors registered with the Medical Council of Ireland in recent years [10, 11, 16]. The scale and trajectory of this national medical workforce crisis is apparent from media reports indicating that no appointable applicants are applying for previously highly sought-after hospital consultant posts in national specialist hospitals [21]. Previous research on this topic by the authors of this paper revealed that doctors emigrate from Ireland mainly due to the difficult working conditions, including the long working hours and uncertain career progression prospects [23].

Ireland collects data on exits from the medical and nursing/midwifery professional registers, which provides a proxy measure for emigration. Health professionals exit these registers most commonly in their 60s due to retirement and death, at any age due to a change in career or a decision to stop practicing medicine temporarily or permanently, or because of emigration. Overall annual exit rates for doctors in 2013 were reported at 6.8 % [16]; however, a better indicator of the likely large scale emigration of doctors from Ireland is the reported annual exit rate of approximately 10 % among doctors aged 25–29 and 30–34 years in 2013 and 2014 [16, 17]. While 9 % of nurses/midwives were officially registered as working abroad [24], this number excludes those who may have removed their names permanently from the nursing register in Ireland upon emigration and those who have emigrated but remain fully registered. Therefore, the available indicators are likely to underestimate the numbers of emigrant nurses/midwives. The United Kingdom, Australia, and Canada ranked as the top three intended destination countries for nurses/midwives in Ireland in 2013 [24], in agreement with media reports indicating these as the most popular destinations for health professionals emigrating from Ireland [25, 26].

Only limited insights have been gained thus far into the reasons underlying the emigration decisions of individuals in HICs [19]. The present study aims to describe how the intentions of emigrant health professionals concerning their planned length of stay in their destination country change over time. The findings from an online survey of emigrant health professionals comparing their intentions at the point of arrival in their destination country with those following a certain period of residency are presented herein. These findings will contribute to a body of work which considers health professional migration from the perspective of the individual health professional and links their migration decisions to wider workforce implications [13, 15, 27, 28]. Furthermore, this study aims to test the feasibility of using online social media platforms (i.e. Facebook) to access and recruit emigrant health professionals. Online social media platforms offer the potential to engage with a wide range of populations in various locations [29], helping to overcome the difficulties associated with the research of emigrant populations [30], and are becoming a common method of accessing populations for research purposes [29, 31–33]. Thus, this paper will also contribute to the body of knowledge on the use of online social media platforms as a research tool to access populations.

## Methods

### Study design and setting

A short, semi-structured survey tool with 23 questions, designed by the authors, was delivered online via Survey Monkey over a 5-week period (beginning in June 2014). The study was reviewed and approved by the Royal College of Surgeons in Ireland Research Ethics Committee.

### Participants

As this paper aimed to explore the intentions of emigrant health professionals (doctors, nurses and midwives), eligible participants were defined as health professionals who had worked in Ireland but who were now working as a health professional in another country. They did not have to have undertaken their primary medical or nursing training in Ireland. Participants were recruited via snowball sampling – a chain-referral method, which is a non-probability sampling method that samples clusters of connected participants [34]. Snowball sampling is an effective method to access a hard-to-reach population in order to undertake formative research [35], which suited the exploratory nature of this study. The recruitment of participants was performed online, mainly through the social networking site, Facebook. The use of online social networking platforms to recruit research participants in the realm of human resources for health is innovative, as most studies of health professionals rely on paper-based recruitment (e.g. [36, 37]) or online recruitment through email (e.g. [38]).

Initially, a number of gatekeepers, namely health professionals living in Ireland in contact with emigrant health professionals and relevant Facebook groups (similar to Bhutta [31]) of Irish health professionals, were identified by the research team. Gatekeepers were contacted by email or by Facebook message/posting and provided with information about the study, including the survey link, and were asked to recruit emigrant doctors, nurses and midwives from their social network to participate in the study, again using email or Facebook contact, or both. Successive sets of respondents recruited potentially eligible health professionals from their own social networks, rapidly extending the reach of the study. Gatekeepers were sent a reminder email 2 weeks into the survey to boost the number of responses. At this point, because the numbers of responding nurses and midwives were much lower than for doctors, a link to the survey was also posted on the Irish Nursing and Midwifery Organisation website to boost recruitment.

### Data sources/measurement

In the survey, respondents were asked to provide non-identifying basic demographic data (e.g. sex, age group, etc.) and to answer a number of open ended questions related to their motivations for emigration and perceptions of the health system in Ireland. The outcomes for this paper are based on responses to three closed response questions. First, the survey sought to ascertain respondents’ intended length of stay abroad at the time of emigration, by asking them “On arrival, how long did you intend to stay here (destination country) for?”, with closed response options: up to 1 year, up to 3 years, up to 5 years, permanently, uncertain, and the open response option ‘other’. Second, it asked about current intentions with respect to staying in their destination country: “Now, how long are you intending to stay here (destination country) for?”, with the same closed responses as above. Third, to ascertain the future intentions of respondents concerning a return to practice in Ireland: “In the future, do you intend to return to practice in Ireland?”, with closed response options: yes, no, maybe, and the open response option ‘other’. The analysis compared (correlated) intended duration of stay as reported on arrival in the destination country with intended duration at the time of survey response.

For doctor respondents, the grades of training defined for this paper are those used in the Irish health system, namely intern (the initial grade for doctors in Ireland immediately after graduating from medical school, completion of which is required as a component of undergraduate medical education in Ireland), Senior House Officer (SHO; comprising the first (initial) stage of specialist training in hospital medicine, usually 2 years in duration), Registrar (usually undertaken between SHO and Specialist Registrar (SpR) grades in hospital medicine; not officially recognised as a training grade although some posts may be assigned as training posts), SpR (comprising the second (higher) stage of specialist training in hospital medicine, up to 7 years in duration depending on the specialty and the final training grade before being eligible to apply for a hospital consultant position), and Consultant (the pinnacle grade in hospital medical training, includes doctors who have completed their SpR training and who can practice independently). General Practitioners (GPs) are designated as either GP trainees (the combined equivalent of SHO and SpR training in general practice, 4 years in duration) or fully qualified GPs. Because of the smaller numbers for nursing/midwifery respondents, the grades defined herein are either student nurse/midwife (undergoing training) or staff nurse/midwife (fully trained). The above three outcome variables were analysed according to the level of grade/seniority of respondent doctors upon emigration from Ireland: junior (Intern, SHO and Registrar) and senior (SpR and Consultant).

**Box 1:** Medical training grades in Ireland

**Intern** – the grade undertaken immediately upon completion of medical school. Completion of this grade is required as a component of under-graduate medical education in Ireland. **Senior House Officer (SHO)** – this grade comprises the first (initial) stage of specialist training in hospital medicine and is usually 2 years in duration. **Specialist Registrar (SpR)** – this grade comprises the second (higher) stage of specialist training in hospital medicine and can be up to 7 years in duration depending on the specialty. **GP Trainee** – this grade comprises the combined equivalent of SHO and SpR training in general practice and is 4 years in duration. **Registrar/Senior Registrar** – this is a grade, usually undertaken between SHO and SpR grades in hospital medicine. This grade is not officially recognised as a training grade although some posts may be assigned as training posts. **Consultant** – this is the pinnacle grade in hospital medical training and comprised those doctors who have completed their SpR training and who can practice independently. **General Practitioner (GP)** – the equivalent grade of a consultant in general practice. **Clinical Attachment/Observer** – this is not an official medical grade but rather a shadowing role that is used to give the doctor familiarity with a new hospital or health system environment. This role is unpaid.	

### Bias

Recruitment into the study was influenced by the social networks of initial gatekeepers, creating a potential selection/sampling bias. However, the cascade effect, whereby those contacted in turn contacted and recruited others, resulting in a rapid increase in the sample size, especially of doctors who had worked in Ireland, would have reduced this initial bias over time. Secondly, interest in and strength of feelings about the topic are likely to have influenced response numbers (volunteerism), perhaps leading to a sample with stronger negative feelings about having had to emigrate from Ireland. On the other hand, those who had retained little interest in the country they had left might also have been under-represented among the responses. Further, respondents were asked to recall their length of stay intentions in their particular destination country at the time they emigrated from Ireland, also raising the risk of recall bias.

### Study size

As discussed, the population size of health professionals emigrating from Ireland is unknown. Previous research has indicated the possible scale of doctor emigration from Ireland, with Ireland being the European country with the highest percentage of doctors practising abroad at 47.5 % [39]. However, a calculation of the exact population size, or even an estimate, is not feasible. In light of this, the study size was determined solely by the number of responses to the survey over the 5-week timeframe. Due to advertisement of the study on Facebook and through email, it is also not possible to determine the number of people who actually clicked on the link to the survey but did not attempt to complete it, similar to other studies using this method of recruitment [31]. Thus, the response rate for this study will actually be a response number purely determined by the number of completed responses.

### Statistical methods

The responses obtained from Survey Monkey were analysed using the Statistical Package for the Social Sciences (SPSS, version 13). Respondent demographic characteristics, emigration history and return intentions are summarised using descriptive statistics (frequencies and percentages). Percentages were calculated excluding missing data unless otherwise stated. Differences between respondents who intended to return and those who did not were assessed using the *χ*^2^ test. The association between an increased length of stay intention and covariates (age, sex, country of training, year of emigration, destination country and grade last worked) was assessed using multiple logistic regression presenting adjusted odds ratios (OR) and 95 % confidence intervals (CI). This analysis was performed only on those respondents who expressed a definite length of stay intention at the time of arrival in their destination country and at the time of survey (i.e. excluding those who were uncertain). Statistical significance at *P* <0.05 was assumed.

## Results

### Respondent characteristics

A total of 556 people accessed the survey, of whom 388 were eligible to participate. Those who were ineligible to participate included health professionals who were working in Ireland (i.e. were not emigrants) and individuals who were not health professionals. The survey was accessed by 216 people (40 % of the total number) in the first 24 h, and 385 (70 %) accessed the survey within 3 days. The rapid recruitment via Facebook in the first few days is similar to that reported in other studies using this method [31, 33].

The respondents included 307 doctors, 73 nurses and 8 midwives. Among the doctor respondents, 138 (51 %) were male, while the nursing/midwifery respondents were mainly female (90 %, *n* = 68; Table 1). The majority of respondents were aged between 25–34 years, (63 % (*n* = 173) of doctor and 36 % (*n* = 27) of nursing/midwifery respondents). Ireland was the most common country of training for all respondents, with 91 % (*n* = 273) of doctor and 80 % (*n* = 63) of nursing/midwifery respondents obtaining their primary health professional training in the country.

**Table 1:** Respondent demographics

		Doctors	Nurses/Midwives
		*n* (%) ^a^	*n* (%) ^a^
Sex			
	Male	138 (51)	8 (11)
	Female	135 (49)	68 (90)
	Total	273 (100)	76 (100)
	Missing	34	5
Age			
	≤24 years	4 (2)	23 (31)
	25–34 years	173 (63)	27 (36)
	35–44 years	91 (33)	17 (23)
	45–54 years	5 (2)	8 (11)
	Total	273 (100)	75 (100)
	Missing	34	6
Country of training			
	Ireland	273 (91)	63 (80)
	United Kingdom	6 (2)	9 (11)
	India	2 (1)	3 (4)
	Sudan	1 (0.3)	0 (0)
	Nigeria	2 (1)	1 (1)
	South Africa	1 (0.3)	1 (1)
	Other	14 (5)	2 (3)
	Total	299 (100)	79 (100)
	Missing	8	2
Year of emigration			
	2012–2014	166 (57)	49 (65)
	2008–2011	104 (36)	19 (25)
	2000–2007	17 (6)	5 (7)
	Up to 1999	2 (1)	2 (3)
	Total	289 (100)	75 (100)
	Missing	18	6
Destination country			
	Australia	92 (33)	23 (32)
	United Kingdom	76 (27)	27 (38)
	USA	57 (20)	2 (3)
	New Zealand	25 (9)	5 (7)
	Canada	26 (9)	1 (1)
	Other	6 (2)	13 (18)
	Total	282 (100)	71 (100)
	Missing	25	10
Grade last worked in Ireland			
	Intern	77 (25)	
	Senior House Officer	56 (18)	
	Registrar	55 (18)	
	Specialist Registrar	85 (28)	
	Consultant	11 (4)	
	General Practitioner	19 (6)	
	Other	3 (1)	
	Total	306 (100)	
	Missing	1	
Student nurse/midwife			13 (16)
Staff nurse/midwife			54 (69)
Other			9 (11)
Total			76 (100)
Missing			5

^a^ Percentages are calculated excluding the missing data

Of the doctor respondents, 93 % (*n* = 270) had emigrated from Ireland since 2008, as had 90 % (*n* = 68) of nursing/midwifery respondents. Thus, the sample of respondents largely represents post-recession emigration from Ireland (Table 1). The most popular destination country for doctor respondents was Australia (33 %, *n* = 92), followed by the United Kingdom (27 %, *n* = 76) and the United States of America (20 %, *n* = 57; Table 1). For nursing/midwifery respondents, the most popular destination country was the United Kingdom (38 %, *n* = 27), followed by Australia (32 %, *n* = 23; Table 1).

The largest number of doctor respondents had last worked at SpR grade (28 %, *n* = 85) in Ireland before emigration, closely followed by intern grade (25 %, *n* = 77), while the majority of nursing/midwifery respondents had worked at staff grade (69 %, *n* = 54).

### Intentions of respondents concerning their length of stay abroad

For doctor respondents, 55 % (*n* = 162) intended to stay for up to 5 years at the time of arrival in their destination country, while only 10 % (*n* = 29) intended to stay permanently (Table 2). At the time of the survey, those intending to remain in their destination country for up to 5 years had fallen to 24 % (*n* = 67), while the percentage who intended to remain permanently had more than tripled to 34 % (*n* = 94).

**Table 2:** Overall responses to the question “How long do you intend to stay here (destination country) for?” on arrival to their destination country and at the time of survey for all respondents

		How long did you intend to stay in your destination country for?						
		≤1 year, %	≤3 years, %	≤5 years, %	Permanently, %	Uncertain, %	Other, %	Total, *n*
Doctors								
	Arrival	18	21	16	10	28	4	282
	Now	2	11	11	34	35	8	281
Nurses/midwives								
	Arrival	32	17	11	12	26	3	76
	Now	1	17	18	26	30	7	76

For nursing/midwifery respondents, 60 % (*n* = 45) intended to stay for up to 5 years on arrival in their destination country, while only 12 % (*n* = 9) intended to stay permanently (Table 2). At the time of survey, the proportion of nursing/midwifery respondents intending to remain for up to 5 years had fallen to 36 % (*n* = 36), while the percentage who intended to remain permanently had more than doubled to 26 % (*n* = 20).

Uncertainty surrounding the intended length of stay in the destination country was consistently above 25 % at both time points (arrival and at the time of survey), and increased slightly over time for both sets of respondents: rising from 28 % (*n* = 80) to 30 % (*n* = 99) for doctor respondents, and from 26 % (*n* = 20) to 30 % (*n* = 23) for nursing/midwifery respondents between the two time points.

**Fig. 1:**
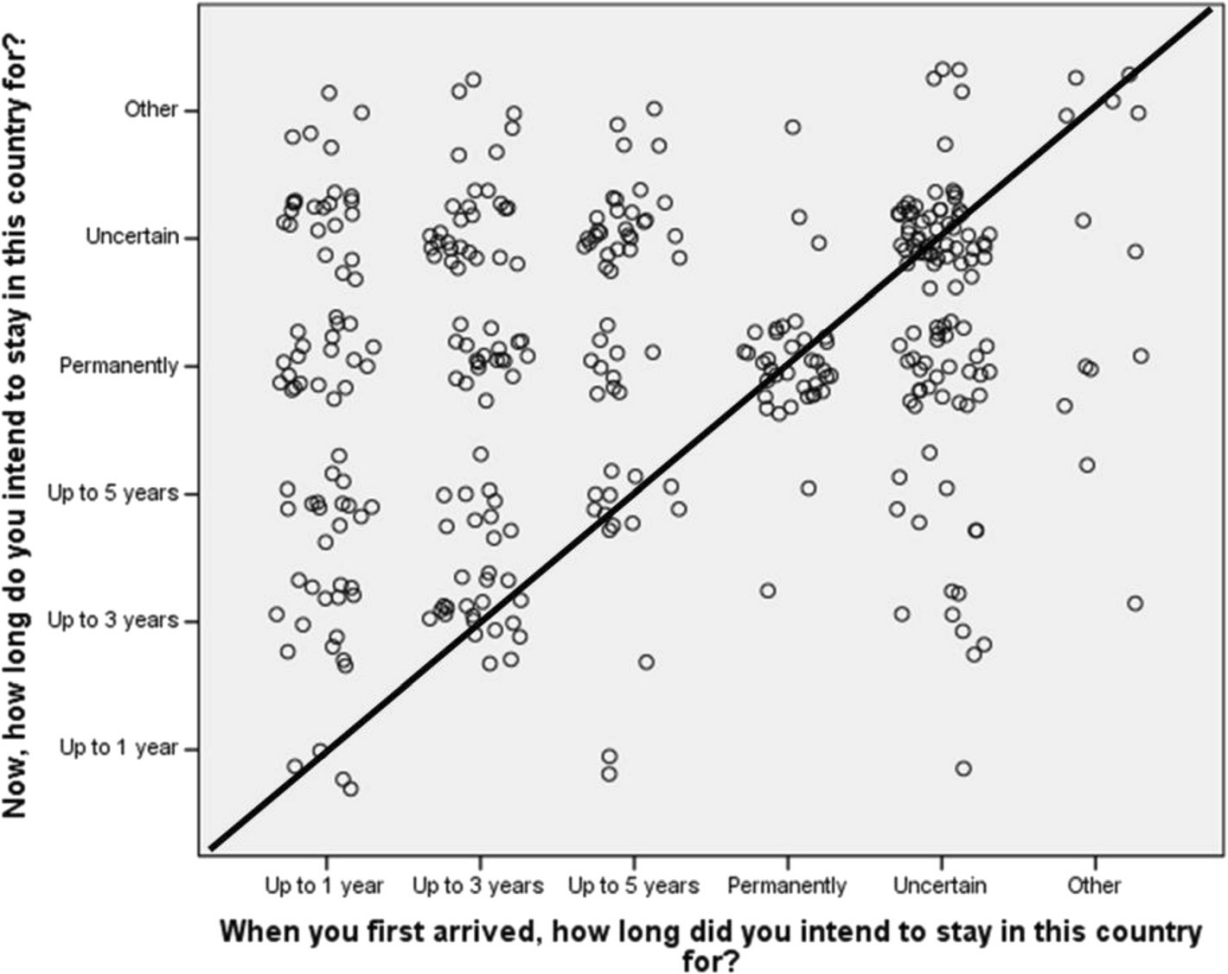
Scatterplot of the results for “When you first arrived, how long did you intend to stay in this country for”? by “Now, how long do you intend to stay in this country for”? The diagonal line represents the line of no change, above the line represents a change in length of stay intention to either a longer time period or uncertainty, and below the line represents a change in length of stay intention to a shorter time period

A trend over time was noted whereby an intended short-term stay was likely to shift towards uncertainty or an intended permanent stay in the destination country, while permanent intentions to stay on arrival were likely to be maintained over time (Fig. 1).

When compared by junior versus senior medical grades, there was a statistically significant difference in intentions to stay at the time of survey (*χ*^2^ = 10.96, *P* = 0.012) but not at the time of arrival. There were no statistically significant differences noted at either time point for nursing and midwifery respondents.

Among the 52 doctors who, at arrival in their destination country, intended to remain there for only up to 1 year, 37 % (*n* = 19) intended to stay permanently at the time of survey, while 21 % (*n* = 11) were uncertain. Among the 29 doctors who intended to stay permanently in their destination country on arrival, 79 % (*n* = 23) still had this intention at the time of survey.

For nurse/midwifery respondents, of the 24 who intended to remain in their destination country for up to 1 year on arrival, 33 % (*n* = 8) were uncertain at the time of survey, while 29 % (*n* = 7) intended to stay for up to 3 years and 25 % (*n* = 6) for up to 5 years; only 4.2 % (*n* = 1) of those respondents intended to remain permanently at the time of survey. From the 9 nursing/midwifery respondents who intended to remain permanently in their destination country at arrival, all respondents retained such intentions at the time of survey.

Investigating the respondents who had made a definite decision concerning their length of stay intentions (i.e. excluding those who were ‘uncertain’ or ‘other’) both at the time of arrival and at the time of survey (*n* = 159; Table 3), Australia as a destination country was a significant predictor of a change in intentions (OR, 2.02) in univariable analysis; however, this significance was not maintained in the adjusted model (Table 3).

**Table 3:** Relationship between change of intention with demographics and variables (*n* = 159)

	Crude OR	Adjusted OR	Adjusted OR CI
Variable			
Age	1.13	0.52	0.19–1.42
Sex	0.58	0.22	0.04–1.19
Year of emigration	0.53	0.42	0.16–1.11
Country of training Ireland	0.00	0.00	0.00
Destination country Australia	2.02	0.25	0.30–2.37
Destination country United Kingdom	1.20	1.90	0.3–12.0

*CI* Confidence interval, *OR* Odds ratio

### Intentions of respondents concerning return to practice in Ireland

At the time of survey, 24 % (*n* = 66) of doctor and 47 % (*n* = 36) of nursing/midwifery respondents intended to return to Ireland in the future (Table 4). Conversely, 33 % (*n* = 91) of doctor and 22 % (*n* = 17) of nursing/midwifery respondents did not intend to return. Finally, 38 % (*n* = 118) of doctor respondents and 26 % (*n* = 21) of nursing/midwifery respondents were uncertain, stating that they would perhaps return to Ireland in the future (Table 4).

**Table 4:** Overall responses to the question “In the future, do you intend to return to Ireland?” for all respondents. Yes, respondents who answered ‘yes – definitely’ and ‘yes – probably’; No, respondents who answered ‘no – definitely’ and ‘no – probably’

	In the future, do you intend to return to Ireland?				
	Yes %	No %	Maybe %	Other %	Total number n
Respondents					
Doctors	24	33	38	1	279
Nurses/midwives	47	22	26	3	76

These data were analysed further by age. For doctor respondents who had a clear intention regarding returning to Ireland, i.e. answered either yes or no, most respondents in both the 25–34 (31 %, *n* = 54) and 35–44 (36 %, *n* = 33) years of age categories did not intend to return to Ireland in the future. However, overall, the majority of respondents in each age category answered ‘maybe’ – 43 % (*n* = 75) for 25–34 years and 42 % (*n* = 38) for 35–44 years – indicating uncertainty but openness to such a move. However, there was no statistically significant difference across age categories (*χ*^2^ = 7.1, *P* = 0.76). Further, a greater percentage of junior doctors (65 %) reported an intention to return to practice in Ireland compared to senior doctors (35 %); however, this difference was not statistically significant (*χ*^2^ = 0.72, *P* = 0.87).

For nursing/midwifery respondents, 61 % of respondents in the under 24 years of age category intended to return to Ireland (*n* = 14), while among the 25–35 years of age category, equal numbers indicated yes and no (33 %, *n* = 9 each). A greater percentage of nurses (96 %) reported an intention to return to practice in Ireland compared to midwives (4 %); however, this difference was not statistically significant (*χ*^2^ = 3.71, *P* = 0.32).

## Discussion

This exploratory study of 388 doctors, nurses and midwives, 87 % of whom had left Ireland between 2008 and 2014, found that short-term emigration intentions had in many cases changed over time to longer term and/or permanent intentions to remain. There was a high level of uncertainty around future plans among those who had originally planned to migrate for a short period; in particular, there was a reduction in the numbers of doctors intending to return to practice in Ireland in the future and a 24 % increase in those intending to stay permanently. An interesting finding among doctor respondents was that, upon arrival in their destination country, there was no significant difference in the length of stay intentions among junior versus senior doctors. However, at the time of survey, a significant difference was observed, with junior doctors more likely to have permanent intentions compared to senior doctors. This difference could be attributed to junior doctors having become established in their destination countries, thus converting temporary intentions to more permanent ones, or to the offer of attractive career progression and training opportunities for early career doctors in the destination countries.

Given Ireland’s commitment to implementing the WHO Global Code, an interesting finding was that, while 89 % (*n* = 336) of respondents were Irish trained, some non-Irish-trained doctors responded to the survey, illustrating the difficulties Ireland faces in striving for health workforce self-sufficiency. Not only does Ireland fail to retain the doctors and nurses it trains, it also fails to retain some of those it recruits from overseas. Indeed, this finding has been confirmed in previous studies of foreign doctors and nurses in Ireland [12, 15].

From a health workforce perspective, the findings illustrate the importance of retaining health professionals within the Irish health system. Once a health professional emigrates, the possibility that their exit may be permanent increases and intentions to return home reduce over time, as reported herein and by Sharma et al. [37] in their study of United Kingdom doctors who had emigrated to New Zealand. However, the high level of uncertainty expressed by respondent emigrant health professionals about their length of stay abroad, along with extensive and strongly articulated views regarding health workforce conditions in Ireland [23], perhaps indicates an opportunity for the Irish health system to re-attract many of these doctors, nurses and midwives.

The method used to access and recruit the respondents has its limitations, in as much as its representativeness cannot be easily estimated due to the lack of a sampling frame. However, its strength lies in its potential for collecting data on large numbers of what is a hard-to-reach population (emigrant health professionals), relatively quickly and with little expense [31]. Source country health workforce planners and policymakers require mechanisms such as these to engage with the emigrant health professionals they have lost. Emigrant health professionals may play a potential role in the source country’s future workforce, provided they can be re-recruited. Further, they can be an important (and currently untapped) source of insight regarding the comparative weaknesses of the source country’s health workforce practices, and indeed its health system, in relation to the destination countries to which these professionals have migrated. This type of information is critical for source countries, like Ireland, if they wish to retain and re-attract the many hundreds of recently emigrated doctors, nurses and midwives. Improving retention and encouraging return would enable Ireland to become less reliant on the recruitment of health professionals from other (often poorer) countries, and more compliant with the WHO Global Code.

### Limitations and conclusion

Our sample of respondents is not representative of all emigrant health professionals from Ireland. As this study was exploratory and tested the feasibility of social networking as a method of recruitment, we were less concerned with the external validity of our results than with the desire to obtain initial information on this hard-to-reach population, which we intend to use for future large-scale projects. In light of this, we do not have a response rate and thus we do not know the size of the sampling frame from which these respondents came, but rather rely on the large number of respondents from a small country as an indicator of the validity of the results. Further, doctors responded to the survey in significantly higher numbers than nurses/midwives, which may reflect that doctors are more active on issues concerning their emigration, e.g. are already mobilised online in a way that nurses/midwives are not.

Recall bias may have been a factor when respondents were recalling their length of stay intentions at the time of arrival in their destination. The covariates available for the bivariate analysis were limited as the survey was not designed for in-depth quantitative analysis. However, the finding that a particular destination country was a significant predictor for a change in length of stay intentions abroad correlates with qualitative data published from this survey revealing that the working conditions in their destination countries were key factors in retaining respondents abroad [23].

Another limitation, as is the case with cross-sectional studies that try to capture migratory flows and intentions over time, is that it will not have captured the behaviour of an unknown number of doctors and nurses who may have left Ireland for some of these destination countries for short periods and then returned to Ireland, or onwards to other countries. Therefore, while the study illustrates the power of social networking for quickly reaching large numbers of health professionals who feel strongly enough to respond to surveys on migration, this is not a substitute for data collection systems that track the movements of migrants longitudinally. Both source and destination country workforce planners need such systems so as to gain a better understanding of health professional migration, informing the development of more effective policies to promote retention and return.

In conclusion, the longer health professionals remain abroad, the less likely they are to return to their home countries. Countries should focus on the implementation of retention strategies if the ‘carousel’ of brain drain is to be interrupted. This would allow source countries to benefit from their investments in training health professionals, rather than relying on international recruitment to meet health system staffing needs. Improved data collection systems are also needed to track the migratory patterns and changing intentions of health professionals. Meanwhile, social networking platforms offer alternative methods of filling this information gap.
